# Academic stress in college students: descriptive analyses and scoring of the SISCO-II inventory

**DOI:** 10.7717/peerj.16980

**Published:** 2024-03-12

**Authors:** Juan-Luis Castillo-Navarrete, Claudio Bustos, Alejandra Guzman-Castillo, Walter Zavala

**Affiliations:** 1 Programa Neurociencias, Psiquiatría y Salud Mental, NEPSAM, Universidad de Concepción, Concepción, Chile; 2 Programa Doctorado Salud Mental, Departamento de Psiquiatría y Salud Mental, Facultad de Medicina, Universidad de Concepción, Concepción, Chile; 3 Departamento de Tecnología Médica, Facultad de Medicina, Universidad de Concepción, Concepción, Chile; 4 Facultad de Ciencias Sociales, Universidad de Concepción, Concepción, Chile; 5 Departamento de Ciencias Básicas y Morfología, Facultad de Medicina, Universidad Católica de la Santísima Concepción, Concepción, Chile; 6 Carrera de Fonoaudiología, Facultad de Ciencias de Salud y Ciencias Sociales, Universidad de Las Americas, Concepción, Chile

**Keywords:** Academic stress, College students, SISCO-II, Stress scoring, Stressors, Physical and psychological reactions, Social behavioural reactions, Coping, Scales

## Abstract

In a competitive and demanding world, academic stress is of increasing concern to students. This systemic, adaptive, and psychological process is composed of stressful stimuli, imbalance symptoms, and coping strategies. The SISCO-II Academic Stress Inventory (SISCO-II-AS) is a psychometric instrument validated in Chile. It evaluates stressors, symptoms, and coping, both individually and globally. For its practical interpretation, a scale is required. Therefore, this study aims to descriptively analyze the SISCO-II-AS and to obtain its corresponding scales. Employing a non-experimental quantitative approach, we administered the SISCO-II-AS to 1,049 second and third-year students from three Chilean universities, with a disproportionate gender representation of 75.21% female to 24.79% male participants. Through descriptive and bivariate analysis, we established norms based on percentiles. For the complete instrument and its subscales, significant differences by sex were identified, with magnitudes varying from small to moderate. For the full instrument and its subscales, bar scale norms by percentile and sex are presented. Each subscale (stressors, physical and psychological reactions, social behavioural reactions, total reaction, and coping) has score ranges defined for low, medium, and high levels. These ranges vary according to the sex of the respondent, with notable differences in stressors and physical, psychological, and social behavioural reactions. This study stands out for its broad and heterogeneous sample, which enriches the representativeness of the data. It offers a comprehensive view of academic stress in college students, identifying distinctive factors and highlighting the importance of gender-sensitive approaches. Its findings contribute to understanding and guide future interventions. By offering a descriptive analysis of the SISCO-II-AS inventory and establishing bar norms, this research aids health professionals and educators in better assessing and addressing academic stress in the student population.

## Introduction

Mental health takes on special relevance in the case of college students. Currently, a growing number of young people are entering higher education seeking academic training and access to better job opportunities. However, this process can be affected by various factors that can trigger mental health problems, negatively impacting performance and retention at university. College students often face multiple demands in terms of academic load, work responsibilities, social life, and family, which can result in increased academic stress (AS) (Barraza-Macías, 2007a; Putwain, 2007). AS gradually increases due to increasing academic demands, pressure for grades, competition, and fear of not meeting expectations, affecting student performance and health (Castillo-Navarrete et al., 2020). Consequently, it has been observed that students facing high levels of AS are more likely to experience anxiety, depression, insomnia, and other mental disorders. These conditions can hurt both a student’s academic performance and general well-being, underscoring the critical need to comprehensively address AS in the educational environment (Castillo-Navarrete et al., 2023a).

The conceptual grasp of ‘academic stress’ is complicated not only by the term’s broad use but also by the diversity of evaluation tools employed. Rather than relying on a single uniform method, researchers utilize a range of instruments such as the Academic Stress Inventory (ASI), Beck Anxiety Inventory (BAI), and the Perceived Stress Scale (PSS), to name a few (Cohen, Kamarck & Mermelstein, 1983; Beck et al., 1988; Polo, Hernández López & Pozo Muñoz, 1996). Each tool brings its lens to the phenomenon, measuring varying dimensions from cognitive and emotional responses to physiological effects, thus contributing to inconsistent outcomes and complicating cross-study comparisons (Barraza-Macías, 2007a; Barraza-Macías, 2007b).

The term ‘academic stress’ is often used broadly, yet its actual scope and limitations may remain elusive. This ambiguity is exacerbated by the variety of terms used to describe the concept, such as ‘student,’ ‘university,’ ‘burnout,’ ‘school,’ and ‘exam stress,’ leading to an unclear conceptualization and an emphasis on stressors and symptoms rather than on a holistic understanding. This lack of a unified definition is acknowledged as a significant obstacle in the literature. For instance, Liu, Ji & Zhang (2023) investigates how general self-efficacy and depression—shaped by factors including gender—can substantially influence academic stress. Such studies underscore the necessity for a more comprehensive and nuanced understanding of this complex phenomenon.

Therefore, a multidimensional approach is needed to understand and address AS. However, the measurement of stress in college students has been based on tools that provide simple measurements with little contextualization. There are also tools to assess stressful situations related to academic, family, and economic aspects. Some examples of these are the Student Life Stress Inventory (Gadzella, 1994), Undergraduate Stress Sources Questionnaire (Blackmore, Tucker & Jones, 2005), Academic Expectations and Stress Inventory (Ang & Huan, 2006) and College Student Stress Scale (Feldt, 2008). In contrast, others have focused on the stressful potential of different academic conditions, such as the Academic Stress Scale of the Academic Stress Questionnaire Cabanach et al. (2010); González Cabanach et al. (2010); Cabanach, Souto-Gestal & Franco (2016).

In this context, Barraza-Macías (2007a) proposed a more comprehensive and processual theoretical model of AS. He defines AS as a systemic, adaptive, and psychological process. This process consists of three moments: (i) the confrontation with demands perceived as stressors; (ii) a systemic imbalance that manifests itself in the form of symptoms; and (iii) a response aimed at re-establishing the balance. Thus, three components are identified in the systemic process: stressful stimuli, symptoms indicating imbalance and coping strategies (Barraza-Macías, 2007b).

Consequently, Barraza-Macías developed the SISCO inventory of academic stress (SISCO-AS). This self-descriptive psychometric instrument is based on a three-factor structure: stressors, symptomatology, and coping (Barraza-Macías, 2007b). In Chile, the SISCO-AS has been used and its psychometric properties have been studied (Guzmán-Castillo et al., 2018; Guzmán-Castillo et al., 2022). In 2020, a modification of the SISCO-AS, the SISCO-II inventory of academic stress (SISCO-II-AS), was introduced. This new version maintains the subscales of stressors and coping. In addition, it identifies two factors in the symptomatology subscale (now called Total Reaction): physical and psychological reactions, and social behavioral reactions (Castillo-Navarrete et al., 2020).

Assessing the level of AS in diverse student populations requires appropriate scales. When studying the psychometric properties of an instrument, several aspects should be considered. These include item analysis, estimating the reliability of the scores, and obtaining evidence of validity (Muñiz & Fonseca-Pedrero, 2019). This last aspect involves the study of dimensionality, the analysis of the differential functioning of the items and, the relationship with external variables. In essence, it refers to the quality of the inferences made from the scores (Muñiz & Fonseca-Pedrero, 2019; Prieto Adánez & Delgado González, 2010). In the case of the SISCO-II-AS, it is necessary to execute the instrument’s baremization. This allows for establishing the necessary cut-off points to interpret the scores obtained, facilitating its practical use (Muñiz & Fonseca-Pedrero, 2019).

Having adequate scales is essential for any instrument intended for a specific study population. The direct score obtained by an individual is not directly interpretable. Therefore, it is necessary to refer it to other individuals within the same normative group. Scales provide information on the position of an individual about the rest of the normative group, assigning a numerical value to each possible score. There are several ways to obtain scales for an instrument, including chronological (age) scales, percentiles, and typical (standardized and/or normalized) scores (Muñiz & Fonseca-Pedrero, 2019; Prieto Adánez & Delgado González, 2010).

In this context, to evaluate diverse student populations about their level of AS, it is necessary to have adequate scales. Therefore, this paper aims to descriptively analyze the SISCO-II-AS and obtain its corresponding scales, based on the sample used when this inventory was reported (Castillo-Navarrete et al., 2020). Following this theoretical framework, the paper details the methods of data collection, participant demographics, and the statistical analysis process utilized. Subsequently, the study results are presented, which address the efficacy of the SISCO-II-AS inventory and establish the corresponding normative scales. The discussion then elucidates the implications of these findings within the current academic milieu, summarizing the study’s pivotal points and proposing avenues for future research.

## Methodology

### Participants

This study, quantitative in nature and non-experimental design, was conducted with a purified sample of 1,049 students. The participants were second and third-year students from three Chilean universities: Universidad de Concepción (UdeC), Católica de la Santísima Concepción (UCSC), and del Desarrollo (UDD). Each student expressed their willingness to participate by signing an informed consent form and a general datasheet. This form was approved by the Scientific Ethical Committee of the Faculty of Medicine of the Universidad de Concepción (No CE 65/2018). Individuals who declared to be under psychological and pharmacological treatment as an important resource to manage their mental health were included. These individuals are part of the student reality and can perform adequately from an academic point of view (Fu & Qiao, 2023; Lieslehto et al., 2023; Tang et al., 2023). Excluding them from the sample could bias the results concerning the university student population as a whole. The gender composition of our sample is representative of the student populations within the faculties and programs to which we had access. A predominance of female participants is consistent with demographic trends observed in similar academic settings, as reported in our previous studies (Castillo-Navarrete et al., 2023a). This demographic characteristic is an important consideration for interpreting the study’s findings within the broader context of academic stress research.

### Instrument

The SISCO-II-AS consists of 33 items. The first item, dichotomous (yes-no), determines whether the respondent continues to answer. The second item identifies the overall self-perception of the level of AS. Eight additional items seek to identify the frequency of environmental demands perceived as stressors. Another 17 items determine the frequency of symptoms or responses to stressful stimuli. Finally, six items seek to identify the frequency of use of coping strategies. The last three sections use a Likert scale (1: never, 5: always). The parts of this instrument can be used in isolation, combined or as a whole (Castillo-Navarrete et al., 2020).

### Procedure

The research team applied this instrument during the student’s second or third year of study. The careers included were Medical Technology, Obstetrics and Childcare, Kinesiology, Phono audiology, Nursing, Dentistry, Chemistry and Pharmacy, and Nutrition and Dietetics. The application of the instrument lasted approximately 10 min and was carried out at the end of 2018.

The data used are available at https://doi.org/10.48665/udec/M6681K

### Statistical analysis

Descriptive analysis was performed using measures of central tendency and dispersion. Categorical variables were analyzed using frequency and percentage. Bivariate and multivariate analyses were performed, comparing groups using each variable separately. Bar norms were established based on percentiles, both for each part of the SISCO-II-AS, individually, combined and as a complete instrument. A significance level of *α* = 0.05 was established. Data were coded in Microsoft Excel and statistical analysis was run with R Studio (R-Project, 2023).

### Handling of missing data

A total of 38 cases had missing data. Specifically, 36 of them had only one missing value, and the remaining two cases had two missing items. Multiple Imputation by Fully Conditional Specification was employed, using the ‘mice’ package in R, with 21 imputed datasets and 25 iterations, ensuring the convergence of the imputation chains. To combine the results of the multiple imputations, Rubin’s methodology was used (Rubin, 1987).

## Results

The sample presented a gender distribution of 75.21% women (789) and 24.79% men (260) (Table 1). According to the university of origin, 49.38% (518) belonged to UdeC, 12.58% (132) to UCSC and 38.04% (399) to UDD. The average age of the students was 21.26 years (SD =1.81), with a range of 18 to 34 years. The majority were in their third to sixth semester, while a small group (33 students) were in their seventh semester or higher.

When evaluating SISCO-II-AS scores by sex, significant differences (*d* = 0.56) were found on the full instrument and subscales. Specifically, the differences are relevant to stressors, physical and physiological reactions, and social behaviour (Table 2). It is important to note that these differences vary in magnitude. They are small for social behavioural reactions (*d* = 0.237) and moderate for stressors (*d* = 0.468) and physical and psychological reactions (*d* = 0.631). It is relevant to consider the moderate size difference in the total reaction (*d* = 0.518). This masks gender differences in both physical and psychological reactions and social behavioural reactions.

No significant differences were observed in the score of the complete instrument according to the university of origin of the participants. Neither, in stressors, physical and psychological reactions, nor in social behavioural reactions (Table 3).

**Table 1:** Distribution of participants according to sex, university and career.

Career	UDEC (518)		UDD (399)		UCSC (132)	
	Men (126)	Women (392)	Men (105)	Women (294)	Men (29)	Women (103)
Nursing	0	0	28	142	13	62
Speech Therapy	9	55	3	33	0	0
Kinesiology	21	33	30	15	0	0
Nutrition and Dietetics	4	75	7	25	0	0
Obstetrics	7	76	0	0	0	0
Dentistry	0	0	37	79	0	0
Chemistry and Pharmacy	45	67	0	0	0	0
Medical Technology	40	86	0	0	16	41
Total	126 (24.32)	392 (75.68)	105 (26.32)	294 (73.68)	29 (21.97)	103 (78.03)
	518 (49.38)		399 (38.04)		132 (12.58)	

**Notes.**

UDEC Universidad de Concepción UDD Universidad del Desarrollo UCSC Universidad Católica de la Santísima Concepción

**Table 2:** Comparison of SISCO-II-EA scores among participants by sex and effect size (Cohen’s d).

Variables	Women		Men		t-statistic	*p*-value	d
	M	SD	M	SD			
Stressors	3.298	0.595	3.010	0.671	t(399.6) = 6.16	**<0.001**	0.468
Physical and psychological reactions	3.210	0.683	2.770	0.739	t(412.5) = 8.48	**<0.001**	0.631
Social behavioural reactions	2.765	0.825	2.567	0.850	t(429.1) = 3.27	**=0.001**	0.237
Total reaction	3.053	0.675	2.698	0.712	t(420.9) = 7.05	**<0.001**	0.518
Coping strategies	3.140	0.632	3.114	0.673	t(417.6) = 0.55	0.585	0.040
Full instrument	3.133	0.474	2.859	0.524	t(405.5) = 7.47	**<0.001**	0.562

**Notes.**

M arithmetic mean SD standard deviation *p*-value probability associated with the t-statistic d Cohen’s d

Bold represents significant differences (*p* ≤ 0.001).

**Table 3:** Comparison of SISCO-II-EA scores between university of origin, and effect size (Eta squared).

Variables	UdeC		UDD		UCSC		*F*-statistical	*p*-value	eta ^2^
	M	SD	M	SD	M	SD			
Stressors	3.257	0.621	3.203	0.634	3.178	0.626	F(2, 1,044) = 1.31	0.271	0.002
Physical and psychological reactions	3.075	0.699	3.130	0.749	3.115	0.736	F(2, 1,044) = 0.68	0.505	0.001
Social behavioural reactions	2.723	0.808	2.690	0.853	2.765	0.894	F(2, 1,044) = 0.44	0.645	0.001
Total reaction	2.951	0.677	2.975	0.720	2.992	0.737	F(2, 1,044) = 0.24	0.787	0.000
Coping strategies	3.141	0.636	3.165	0.639	3.011	0.667	F(2, 1,044) = 2.92	0.055	0.006
Full instrument	3.067	0.487	3.07	0.528	3.043	0.509	F(2, 1,044) = 0.15	0.861	0.000

**Notes.**

UDEC Universidad de Concepción UDD Universidad del Desarrollo UCSC Universidad Católica de la Santísima Concepción M arithmetic mean SD standard deviation *p*-value probability associated with *F* statistic eta^2^ Eta squared

**Figure 1:**
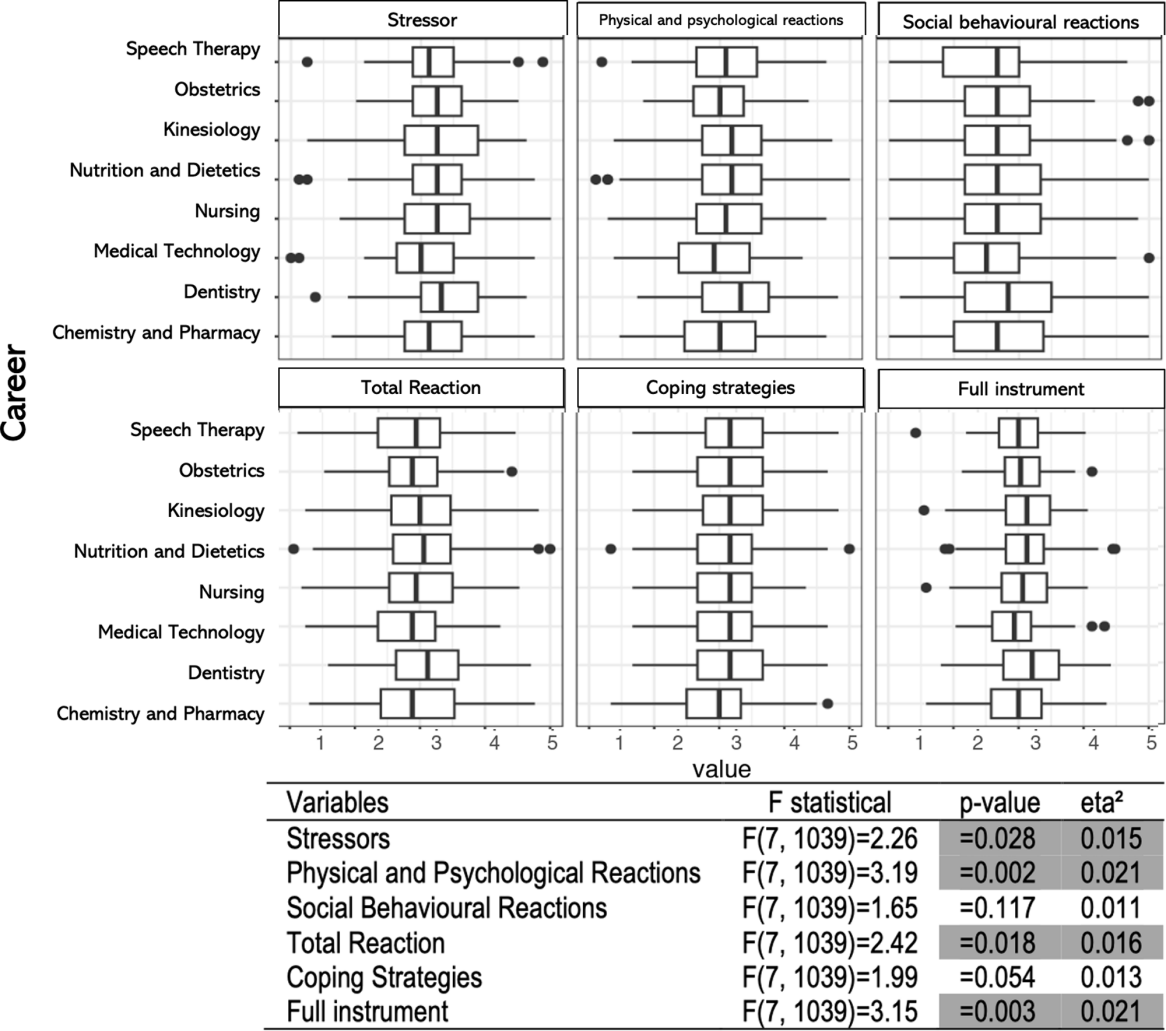
Differences between the careers by the university of origin. Show differences founded between the careers according to the university of origin.

When evaluating the differences between careers of origin (Fig. 1), small differences were found for the complete instrument (eta2 = 0.021). Likewise, small differences were observed in the subscales of stressors, physical and psychological reactions, and coping. However, no significant differences were found for social behavioural reactions, with effect sizes close to 0.01, and very similar for all variables. Figure 1 shows that Dentistry has the highest values in stressors, physical and psychological reactions, as well as social behavioural reactions. On the other hand, Medical Technology has the lowest scores in stressors and physical and psychological reactions. As regarding Chemistry and Pharmacy, show the lowest scores in coping strategies.

Of the total number of participants, 6.96% (73) reported receiving psychological treatment, and of these, 37% (Castillo-Navarrete et al., 2023b) were taking medication. In the context of obtaining the SISCO-II-AS scale in college students, it was decided to include those receiving psychological and pharmacological treatment. These individuals are part of the student reality and can perform academically (Fu & Qiao, 2023; Lieslehto et al., 2023; Tang et al., 2023). This group of participants showed moderate differences in the score of the full instrument (*d* = 0.614). When analyzing the subscales individually, significant differences were found in all except coping (Table 4). Specifically, a small difference was observed in stressors (*d* = 0.225). The differences were greater in physical and psychological reactions (*d* = 0.766) and social behavioural reactions (*d* = 0.641).

**Table 4:** Comparison of SISCO-II-AS scores between participants with and without psychological and pharmacological therapy, and effect size (Cohen’s d).

Variables	With therapy		Without therapy				
	M	SD	M	SD	t-statistic	*p*-value	d
Stressors	3.358	0.685	3.217	0.621	t(79.2) = 1.71	0.092	0.225
Physical and psychological reactions	3.606	0.678	3.063	0.712	t(82.4) = 6.58	**<0.001**	0.766
Social behavioural reactions	3.208	0.821	2.679	0.825	t(81.3) = 5.30	**<0.001**	0.641
Total reaction	3.466	0.641	2.928	0.691	t(83.1) = 6.88	**<0.001**	0.783
Coping strategies	3.002	0.643	3.144	0.641	t(81.2) = 1.81	0.074	0.220
Full instrument	3.348	0.476	3.044	0.496	t(82.2) = 5.25	**<0.001**	0.614

**Notes.**

M arithmetic mean SD standard deviation *p*-value probability associated with the *t*-statistic d Cohen’s d

Bold represents significant differences (*p* ≤ 0.001).

**Figure 2:**
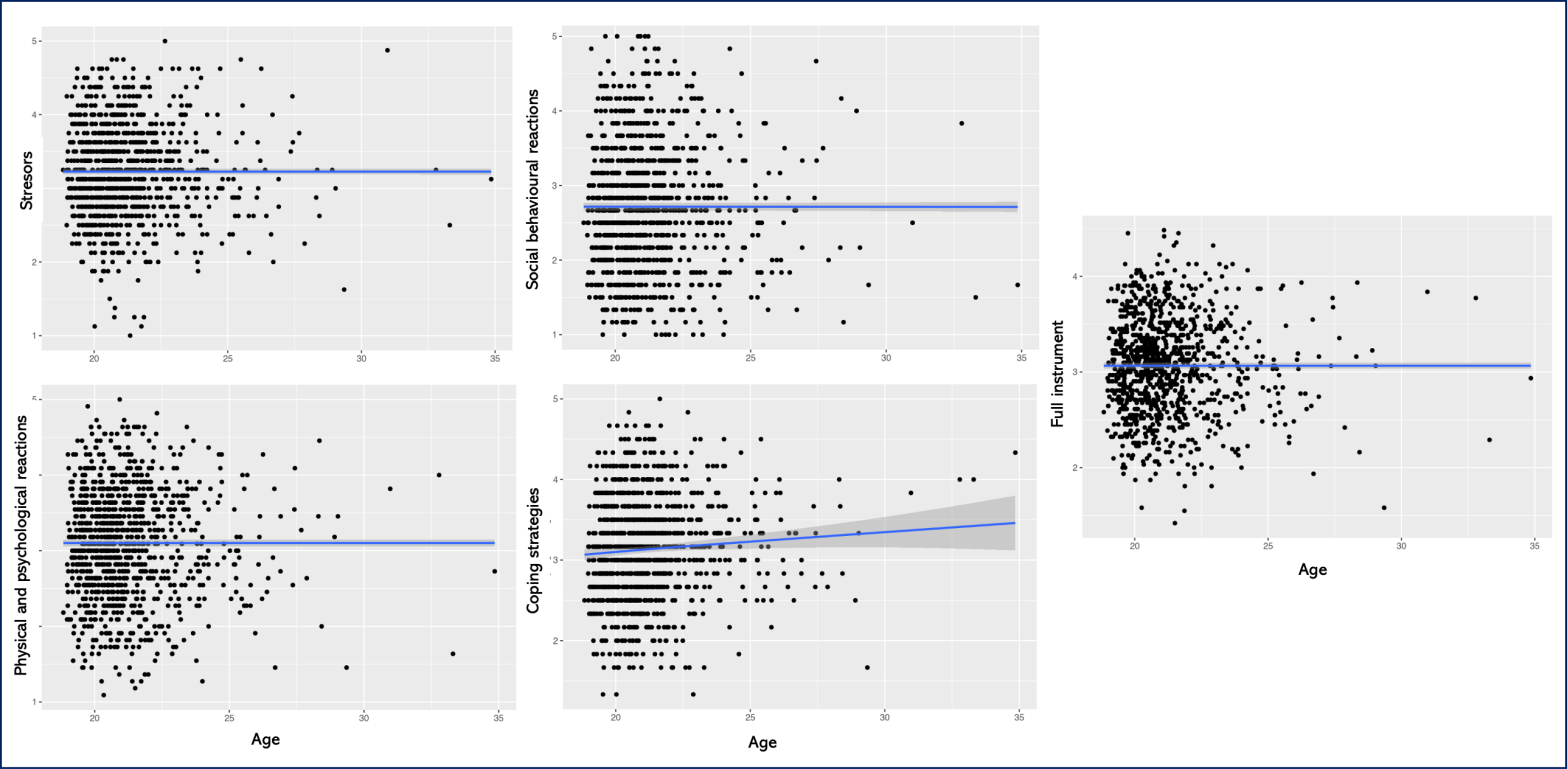
Scatter plot showing the relationship between age and the SISCO-II-AS. Scatter plot showing the relationship between age and the subscales of stressors, physical and psychological reactions, social behavioural reactions, total reaction, coping strategies and for the whole instrument.

When examining the relationship between the age of the participants and the instrument, a linear relationship close to *r* = 0 was mostly observed (Fig. 2). However, the coping subscale showed a significant relationship with age, although of a small magnitude (*r* = 0.09, *p* < 0.01).

Considering that differences by university and career are minimal, differences by age are insignificant, and differences in stress level are predictable in individuals on psychological and pharmacological therapy, sex is identified as the relevant variable in setting norms. There is no clear explanation for why a group should show significant differences in the subscales or the entire instrument. Therefore, norms are established for each subscale and the full instrument using percentile <25th percentile, 25th–50th percentile and >50th percentile cut points for low, medium, and high levels, respectively (Gutiérrez et al., 2016). A summary of the norms established for the full instrument and its subscales is presented in Table 5. For a more detailed analysis, a breakdown by percentile and gender is available as supplementary material.

**Table 5:** Summary of the standards set for SISCO-II-AS (full instrument and its subscales).

Variable	SISCO-II-AS score					
	Women			Men		
	Low AS	Medium AS	High AS	Low AS	Medium AS	High AS
Stressors	≤23	24–29	≥ 30	≤20	21–27	≥28
Physical and psychological reactions	≤30	31–40	≥ 41	≤25	26–35	≥36
Social behavioural reactions	≤12	13–19	≥ 20	≤11	12–18	≥19
Total reaction	≤44	45–59	≥ 60	≤38	39–53	≥54
Coping strategies	≤16	17–21	≥ 22	≤16	17–21	≥22
Full instrument	≤86	87–107	≥ 108	≤78	79–98	≥99

**Notes.**

Low AS Low level of AS given by centiles 0–25 Medium AS Medium level of AS given by centiles 25–75 High AS High level of AS given by centiles 75–100

For the stressors subscale, it is considered low level if women score ≤ 23 and men ≤ 20. The mean score is between 24 and 29 for women, and between 21 and 27 for men. A high score corresponds to ≥ 30 for women and ≥ 28 for men. In the physical and psychological reactions subscale, a high score is reached with ≥ 41 for women and ≥ 36 for men. The intermediate score varies between 31 and 40 for women, and between 26 and 35 for men. Thus, ≤ 30 for women and ≤ 25 for men indicate a low level on this subscale. For social behavioural reactions, a high score is ≥ 20 for women and ≥ 19 for men. A medium score for women is in the range of 13 to 19 points, and for men, it is in the range of 12 to 18 points. Therefore, ≤ 12 for women and ≤ 11 for men indicates a low level in this subscale.

In the total score of the total reaction subscale, which includes physical and psychological reactions and social behavioural reactions (Castillo-Navarrete et al., 2020), a high level is considered when women score ≥ 60 points and men score ≥ 54 points. A medium level is between 45 and 59 points for women, and between 39 and 53 points for men. On the other hand, ≤ 44 for women and ≤ 38 for men will correspond to a low level. In coping strategies, a high score is established for women and men scoring ≥ 22 points. A mild score is considered for those with scores between 17 and 21, while a low score corresponds to ≤ 16.

When evaluating the full instrument, for women it is established that a high level of AS is reached with ≥ 108 points (Table 5). A medium level is in the range of 87 to 107 points, while ≤ 86 points corresponds to a low level. For men, a high level of AS is achieved with ≥ 99 points. A medium level ranges from 79 to 98 points, and ≤ 78 points indicates a low level.

Summarising the key findings, our analysis identified significant differences in several variables. Significant differences by sex were found for stressors, physical and physiological reactions, social behaviour, and the entire instrument, which reaffirms the importance of considering sex when studying academic stress. In addition, the scale of scores on the SISCO-II-AS and its sub-scales is essential for an accurate and contextual interpretation of academic stress levels, allowing for meaningful comparisons within the student population.

## Discussion

AS is a common experience in the lives of college students, potentially leading to negative physical and mental health consequences. Adequate assessment of academic stress is crucial to identify students at risk and provide the necessary intervention. The present study aimed to descriptively analyse the SISCO-II-AS and to obtain its corresponding scales. The instrument was administered before the COVID-19 pandemic (face-to-face academic activities). During the pandemic, academic conditions underwent significant changes. However, with the return to traditional academic activities, a proper objectification of academic stress is essential.

The SISCO-II-AS has strong psychometric properties (Castillo-Navarrete et al., 2020; Guzmán-Castillo et al., 2022; Castillo-Navarrete et al., 2023b). These ensure the reliability and validity of the instrument in measuring academic stress in college students. To interpret the scores obtained in the instrument, the respective bar norms are required. These set percentile cut-off points for categorising AS levels into low, medium, and high (Table 5 and Supplemental Material). This provides a clear guide for the interpretation of the scores. The instrument also takes into account gender differences in its scores. This reflects the well-documented differences in stress response between men and women (Goldfarb, Seo & Sinha, 2019; Graves et al., 2021; Kuhn et al., 2023; Matud, 2004). These gender differences in the stress response are observed at biological, psychological, and social levels (Tamres, Janicki & Helgeson, 2002).

The present study shows several important implications for the analysis of AS. First, it is notable that the gender distribution of the sample is skewed towards women, who represent 75.21% of the participants. Our sample, with a higher number of women, specifically reflects the demographics of accessible health faculties, not a global trend. The selection was based on the availability and cooperation of such faculties, which explains the absence of majors such as engineering. The detection of significant differences in the total scores of the SISCO-II-AS and its subscales according to the gender of the participants is consistent with existing literature, which suggests that women and men may experience and manage stress differently (Matud, 2004). Specifically, significant differences were found in stressors, physical and psychological reactions, and social behavioural reactions. The observed gender differences in SISCO-II-AS scores can be explained by several reasons. For example, on the stressors subscale, women have a low-stress score if they score 23 or less, while for men the threshold is 20. It is plausible to posit that women might be more susceptible to certain academic stressors or have a greater willingness to report their stressful experiences. These gender differences may be attributable to biological, psychological, and socio-cultural factors, and highlight the need to take gender into account in the assessment and management of AS.

The findings also reveal that, despite significant differences in physical and psychological reactions as well as in social behavioural reactions, no significant differences were observed on the coping strategies subscale according to gender. This result is in line with studies suggesting that gender differences in coping may be less pronounced in specific contexts, such as academia (Tamres, Janicki & Helgeson, 2002). However, one should not overlook what has already been reported regarding the weakness of the coping sub-scale (Guzmán-Castillo et al., 2022). Therefore, it is important not to forget that these interpretations require further research to fully understand the neuropsychophysiological causes underlying the stress response.

Regarding the university of origin, no significant differences were found in the scores of the whole instrument or the subscales. When disaggregated by degree, it stands out that dental students obtained the highest scores on the SISCO-II-AS. However, this does not differ from what is reported in the literature (Avramova, 2023; Owczarek, Lion & Radwan-Oczko, 2020). Dental students face high levels of stress due to the rigorous demands of their academic and practical training. In addition, they are under pressure when dealing with patients and facing complex clinical situations from the early stages of their training. Fear of making mistakes and long hours of study and practice can also increase stress. In addition, dentistry involves a significant financial investment, which adds additional pressure and anxiety (Avramova, 2023; Owczarek, Lion & Radwan-Oczko, 2020).

In our study, medical technology students exhibited lower scores in stressors and physical and psychological reactions. This trend might be attributed to the holistic nature of their training, which not only emphasizes a solid grounding in ethical and scientific-technical aspects but also incorporates a significant practical and applied work component. This practical approach could help students connect their learning with real-life situations, potentially reducing anxiety and stress related to theoretical learning and exams. In contrast, students in chemistry and pharmacy displayed the lowest scores in coping strategies. This finding suggests that, while students across different majors may experience comparable levels of stress, the coping strategies they employ might differ. This is consistent with the literature indicating that coping is a dynamic process influenced by a variety of factors, including the educational environment (Folkman & Moskowitz, 2004).

The present study also reveals that students undergoing psychological and pharmacological treatment have higher levels of stress. This underlines the importance of considering mental health in the assessment and management of AS. This finding supports research demonstrating the high prevalence of mental health problems among college students (Auerbach et al., 2018).

It is crucial to acknowledge certain limitations of this study. Primarily, our sample is predominantly composed of students from specific universities and fields of study, which could potentially limit the generalizability of our results to broader student populations. The observed gender imbalance is reflective of the demographic makeup of the faculties and programs accessed, consistent with patterns noted in our previous research (Castillo-Navarrete et al., 2023a). This may affect the presentation and metrics of academic stress and associated biomarkers like BDNF and the percentage of global DNA methylation. We’ve attempted to mitigate these issues through gender-stratified analyses, but we recommend interpreting our findings with care, particularly when extending them to more gender-balanced groups. Secondly, the cross-sectional design of our study restricts our capacity to establish causality. While correlations between AS and various factors have been identified, the directional nature of these relationships remains unclear. Thirdly, the reliance on self-reported measures, such as the SISCO-II-AS inventory, introduces the possibility of response biases, including the tendency toward socially desirable answers, which might skew the results. Lastly, the study does not account for external variables that could significantly impact AS, such as familial, financial, or environmental factors, nor does it examine the nuances of individual learning experiences and workloads. The exclusion of these elements could constrain a more nuanced understanding of AS. Future research should aim to integrate these considerations to enhance our comprehension of the academic stress landscape.

This study exhibits crucial strengths in terms of its relevance and applicability. First, it employs a large and diverse sample, composed of students from different disciplines, enhancing the representativeness of the data. This broad representation reinforces the generalisability of the results to a large and varied student population. In addition, it provides a comprehensive and valuable insight into the prevalence and dynamics of academic stress among college students. In this regard, it identifies differentiating factors according to the field of study and gender. Furthermore, the differential impact of AS on men and women highlights the need for supportive approaches that take these gender differences into account. Although the study has limitations, its findings contribute significantly to the understanding of AS in the university context, providing useful clues for future research and guiding intervention strategies aimed at improving students’ mental health and academic performance.

In conclusion, this study contributes to the field of AS research by providing a descriptive analysis of the SISCO-II-AS inventory and establishing norms for its interpretation. These norms are useful for health professionals, educators and other specialists working with college students, as they allow them to more accurately assess the level of AS and design appropriate interventions to promote student well-being.

##  Supplemental Information

10.7717/peerj.16980/supp-1 Supplemental Information 1 Breakdown by percentile and gender for SISCO-II-AS and its sub scales

10.7717/peerj.16980/supp-2 Supplemental Information 2 Codebook

10.7717/peerj.16980/supp-3 Supplemental Information 3 Dataset

10.7717/peerj.16980/supp-4 Supplemental Information 4 Procedure used for the improvement of writing in English Some sections of this article were written with the help of the GPT-4 AI model.

## Additional Information and Declarations

### Data Availability

The following information was supplied regarding data availability: The data is available at Repositorio de Datos—UdeC: Castillo-Navarrete, Juan-Luis; Bustos, Claudio; Guzmán-Castillo; Alejandra; Zavala, Walter, 2023, “Academic Stress in College Students: Descriptive Analyses and Scoring of the SISCO-II Inventory”, https://doi.org/10.48665/udec/M6681K, Repositorio de Datos—UdeC, V2.

## References

[ref-1] Ang RP, Huan VS (2006). Academic expectations stress inventory. Educational and Psychological Measurement.

[ref-2] Auerbach RP, Mortier P, Bruffaerts R, Alonso J, Benjet C, Cuijpers P, Demyttenaere K, Ebert D, Green J, Hasking P, Murray E, Nock M, Pinder-Amaker S, Sampson N, Stein D, Vilagut G, Zaslavsky A, Kessler R (2018). WHO world mental health surveys international college student project: prevalence and distribution of mental disorders. Journal of Abnormal Psychology.

[ref-3] Avramova N (2023). Self-perceived sources of stress and burnout determinants in dentistry - a systematic review. Galician Medical Journal.

[ref-4] Barraza-Macías A (2007a). Academic stress: A state of the art. Psychology Magazine Cientifica.com.

[ref-5] Barraza-Macías A (2007b). Psychometric properties of the SISCO Stress Inventory academic. PsicologiaCientifica.com Magazine.

[ref-6] Beck AT, Epstein N, Brown G, Steer RA (1988). An inventory for measuring clinical anxiety: psychometric properties. Journal of Consulting and Clinical Psychology.

[ref-7] Blackmore AM, Tucker B, Jones S (2005). Development of the undergraduate sources of stress questionnaire. International Journal of Therapy and Rehabilitation.

[ref-8] Cabanach RG, Souto-Gestal A, Franco V (2016). Academic Stressors Scale for the evaluation of academic stressors in university students. Magazine Ibero-American Psychology and Health.

[ref-9] Cabanach RG, Valle A, Rodríguez S, Piñeiro I, Freire C (2010). Coping Scale of Academic Stress (A-CEA). Ibero-American Journal of Psychology and Health.

[ref-10] Castillo-Navarrete JL, Bustos C, Guzman-Castillo A, Vicente B (2023a). Increased academic stress is associated with decreased plasma BDNF in Chilean college students. PeerJ.

[ref-11] Castillo-Navarrete JL, Guzmán-Castillo A, Bustos C, Rojas R (2023b). Peripheral brain-derived neurotrophic factor (BDNF) and salivary cortisol levels in college students with different levels of academic stress. Study protocol. PLOS ONE.

[ref-12] Castillo-Navarrete J, Guzmán-Castillo A, Bustos NC, Zavala SW, Vicente PB (2020). Psychometric Properties of the SISCO-II Academic Stress Inventory. Magazine Iberoamerican Diagnosis and Assessment and Psychological Assessment.

[ref-13] Cohen S, Kamarck T, Mermelstein R (1983). A global measure of perceived stress. Journal of Health and Social Behavior.

[ref-14] Feldt RC (2008). Development of a brief measure of college stress: the college student stress scale. Psychological Reports.

[ref-15] Folkman S, Moskowitz JT (2004). Coping: pitfalls, and promise. Annual Review of Psychology.

[ref-16] Fu M, Qiao W (2023). Analysis and countermeasures of psychological characteristics in college students’ psychological education based on artificial intelligence. Applied Artificial Intelligence.

[ref-17] Gadzella BM (1994). Student-life stress inventory: identification of and reactions to stressors. Psychological Reports.

[ref-18] Goldfarb EV, Seo D, Sinha R (2019). Sex differences in neural stress responses and correlation with subjective stress and stress regulation. Neurobiology of Stress.

[ref-19] González Cabanach R, Fernández Cervantes R, González Doniz L, Freire Rodríguez C (2010). Estresores académicos percibidos por estudiantes universitarios de ciencias de la salud. Fisioterapia.

[ref-20] Graves BS, Hall ME, Dias-Karch C, Haischer MH, Apter C (2021). Gender differences in perceived stress and coping among college students. PLOS ONE.

[ref-21] Gutiérrez S, Sanz J, Espinosa R, Gesteira C, García-Vera MP (2016). The Scale of Marlowe-Crowne Social Desirability: scales for the general population Spanish and development of a short version. Anales de Psicología/Annals of Psychology.

[ref-22] Guzmán-Castillo A, Bustos C, Zavala W, Navarrete JLC (2022). SISCO inventory of academic stress. Psychological Therapy.

[ref-23] Guzmán-Castillo AG, Saez K, Perez C, Castillo Navarrete JL (2018). Validity and reliability of SISCO inventory of academic stress among health students in Chile. Journal of Pakistan Medical Association.

[ref-24] Kuhn L, Noack H, Wagels L, Prothmann A, Schulik A, Aydin E, Nieratschker V, Derntl B, U Habel (2023). Sex-dependent multimodal response profiles to psychosocial stress. Cerebral Cortex.

[ref-25] Lieslehto J, Tiihonen J, Lähteenvuo M, Mittendorfer-Rutz E, Tanskanen A, Taipale H (2023). Association of pharmacological treatments and real-world outcomes in borderline personality disorder. Acta Psychiatrica Scandinavica.

[ref-26] Liu X, Ji X, Zhang Y (2023). Trajectories of college students’ general self-efficacy, the 456 related predictors, and depression: a piecewise growth mixture modeling 457 approach. Heliyon.

[ref-27] Matud MP (2004). Gender differences in stress and coping styles. Personality and Individual Differences.

[ref-28] Muñiz J, Fonseca-Pedrero E (2019). Ten steps for test development. Psicothema.

[ref-29] Owczarek JE, Lion KM, Radwan-Oczko M (2020). Manifestation of stress and anxiety in the stomatognathic system of undergraduate dentistry students.

[ref-30] Polo A, Hernández López JM, Pozo Muñoz C (1996). Academic stress assessment in university students. Anxiety and Stress.

[ref-31] Prieto Adánez G, Delgado González AR (2010). Reliability and validity. Roles of the psychologist. Issue dedicated to: Methodology at the service of the psychologist.

[ref-32] Putwain D (2007). Researching academic stress and anxiety in students: some methodological considerations. British Educational Research Journal.

[ref-33] R-Project (2023). https://www.r-project.org/.

[ref-34] Rubin DB (1987). Multiple imputation for nonresponse in surveys.

[ref-35] Tamres LK, Janicki D, Helgeson VS (2002). Sex differences in coping behavior: a meta-analytic review.

[ref-36] Tang H, Dai M, Du X, Hung JL, Li H (2023). Understanding college students’ cognitive engagement in online collaborative problem-solving: a multimodal data analysis.

