# Supplementary material for: Academic stress in college students: descriptive analyses and scoring of the SISCO-II inventory
Source: PeerJ. 2024 Mar 12;12:e16980. doi: 10.7717/peerj.16980 (PMC10941763; doi:10.7717/peerj.16980)
Supplement: Supplemental Information 1 [file peerj-12-16980-s001.pdf]

	Stressors			
			Score	
		percentiles	Women	Men
Low AS	0	00 - 05	≤18	≤15
	1	05 - 10	19 - 20	16 - 17
	2	10 - 15	21	18
	3	15 - 20	22	19
	4	20 - 25	23	20
Medium AS	5	25 - 30	------	21
	6	30 - 35	24	22
	7	35 - 40	25	-----
	8	40 - 45	------	23
	9	45 - 50	26	24
	11	55 - 60	27	25
	12	60 - 65	28	-----
	13	65 - 70	------	26
	14	70 - 75	29	27
High AS	15	75 - 80	30	28
	16	80 - 85	31	29
	17	85 - 90	32	30
	18	90 - 95	33 - 34	31 - 32
	19	95 - 100	35 - 40	33 - 40

	Physical and Psychological Reactions			
			Score	
		percentiles	Women	Men
Low AS	0	00 - 05	≤22	≤17
	1	05 - 10	23 - 25	18 - 20
	2	10 - 15	26 - 27	21 - 22
	3	15 - 20	28	23
	4	20 - 25	29 - 30	24 - 25
Medium AS	5	25 - 30	31	26
	6	30 - 35	32	27
	7	35 - 40	33	28
	8	40 - 45	34	29
	9	45 - 50	35	30
	10	50 - 55	36	31
	11	55 - 60	37	32
	12	60 - 65	38	33
	13	65 - 70	39	34
	14	70 - 75	40	35
High AS	15	75 - 80	41	36
	16	80 - 85	42 - 43	37 - 38
	17	85 - 90	44 - 45	39 - 40
	18	90 - 95	46 - 47	41 - 43
	19	95 - 100	48 - 55	44 - 55

	Social Behavioural Reactions			
			Score	
		percentiles	Women	Men
Low AS	0	00 - 05	≤9	≤8
	1	05 - 10	10	9
	2	10 - 15	11	10
	3	15 - 20	12	11
	4	20 - 25	------	------
Medium AS	5	25 - 30	13	12
	6	30 - 35	14	13
	8	40 - 45	15	14
	9	45 - 50	16	15
	11	55 - 60	17	16
	12	60 - 65	18	17
	14	70 - 75	19	18
High AS	15	75 - 80	20	19
	16	80 - 85	21	20
	17	85 - 90	22 - 23	21 - 22
	18	90 - 95	24 - 25	23 - 24
	19	95 - 100	26 - 30	25 - 30

	Total Reaction			
			Score	
		percentiles	Women	Men
Low AS	0	00 - 05	≤32	≤26
	1	05 - 10	33 - 36	27 - 30
	2	15 - 20	37 - 39	31 - 33
	3	15 - 20	40 - 42	34 - 36
	4	20 - 25	43 - 44	37 - 38
Medium AS	5	25 - 30	45	39
	6	30 - 35	46 - 47	40 - 41
	7	35 - 40	48	42
	8	40 - 45	49 - 50	43 - 44
	9	45 - 50	51	45
	10	50 - 55	52 - 53	46 - 47
	11	55 - 60	54	48
	12	60 - 65	55 - 56	49 - 50
	13	65 - 70	57	51
	14	70 - 75	58 - 59	52 - 53
High AS	15	75 - 80	60 - 61	54 - 55
	16	80 - 85	62 - 63	56 - 57
	17	85 - 90	64 - 66	58 - 60
	18	90 - 95	67 - 70	61 - 64
	19	95 - 100	71 - 85	65 - 85

	Coping Strategies			
			Score	
		percentiles	Women	Men
Low AS	0	00 - 05	≤12	≤12
	1	05 - 10	13	13
	2	10 - 15	14	14
	3	15 - 20	15	15
	4	20 - 25	16	16
Medium AS	5	25 - 30	------	------
	6	30 - 35	17	17
	7	35 - 40	------	------
	8	40 - 45	18	18
	9	45 - 50	------	------
	10	50 - 55	19	19
	11	55 - 60	------	------
	12	60 - 65	20	20
	13	65 - 70	------	------
	14	70 - 75	21	21
High AS	15	75 - 80	22	22
	17	85 - 90	23	23
	18	90 - 95	24 - 25	24 - 25
	19	95 - 100	26 - 30	26 - 30

	Full instrument			
			Score	
		percentiles	Women	Men
Low AS	0	00 - 05	≤72	≤63
	1	05 - 10	73 - 77	64 - 69
	2	10 - 15	78 - 81	70 - 72
	3	15 - 20	82 - 84	73 - 75
	4	20 - 25	85 - 86	76 - 78
Medium AS	5	25 - 30	87 - 89	79 - 80
	6	30 - 35	90 - 91	81 - 82
	7	35 - 40	92 - 93	83 - 84
	8	40 - 45	94 - 95	85 - 86
	9	45 - 50	96 - 97	87 - 88
	10	50 - 55	98 - 99	89 - 90
	11	55 - 60	100	91 - 92
	12	60 - 65	101 - 102	93 - 94
	13	65 - 70	103 - 105	95 - 96
	14	70 - 75	106 - 107	97 - 98
High AS	15	75 - 80	108 - 109	99 - 101
	16	80 - 85	110 - 112	102 - 104
	17	85 - 90	113 - 116	105 - 107
	18	90 - 95	117 - 121	108 - 113
	19	95 - 100	122 - 139	114 - 139
