# Supplementary material for: Academic stress in college students: descriptive analyses and scoring of the SISCO-II inventory
Source: PeerJ. 2024 Mar 12;12:e16980. doi: 10.7717/peerj.16980 (PMC10941763; doi:10.7717/peerj.16980)
Supplement: Supplemental Information 2 [file peerj-12-16980-s002.pdf]

Variable	Description	Remarks
.ID	Participant ID	
-9	Missing values	
.Sex<br>DateBirrth	Sex: 0=F;1=M<br>Date of birth	 dd/mm/yyyy
DateAss	Date of assessment	dd/mm/yyyy
Age	Age assessment	years
Univ	University	1: UdeC; 2: UDD; 3: UCSC
Car	Career	1001: UdeC Medical Technology common plan; 1002: UdeC Medical Technology mention in Clinical Bioanalysis, Haematology and Transfusion Medicine; 1003: UdeC Medical Technology mention in Ophthalmology and Optometry; 1004: UdeC Medical Technology mention in Morphophysiopathology and Cytodiagnosis; 1005: UdeC Medical Technology, mention in Imaging and Medical Physics; 1006: UdeC Obstetrics; 1007: UdeC Kinesiology; 1008: UdeC Speech Therapy; 1009: UdeC Chemistry and Pharmacy; 1010: UdeC Nutrition and Dietetics; 2001: UDD Speech Therapy; 2002: UDD Kinesiology; 2003: UDD Nursing; 2004: UDD Nutrition and Dietetics; 2005: UDD Dentistry; 3001: UCSC Medical Technology mention in Clinical Bioanalysis, Hematology and Transfusion Medicine; 3002: UCSC Medical Technology mention in Ophthalmology and Optometry; 3003: UCSC Medical Technology common plan; 3004: UCSC Nursing
Sem	Semester assessment	
	SISCO-II academic stress inventory	
P1	1:Yes 0:No	
P2	Number	
P3_1	Overload of homework and academic work	1. Never<br>2. Rarely<br>3. Sometimes<br>4. Most of the time<br>5. Always
P3_2	The personality and character of teachers	1. Never<br>2. Rarely<br>3. Sometimes<br>4. Most of the time<br>5. Always
P3_3	Teachers' evaluations (exams, essays, research papers, etc.)	1. Never<br>2. Rarely<br>3. Sometimes<br>4. Most of the time<br>5. Always
P3_4	The type of work teachers ask you to do (map consultation, worksheets, essays, concept maps, etc.).	1. Never<br>2. Rarely<br>3. Sometimes<br>4. Most of the time<br>5. Always
P3_5	Failure to understand the topics covered in class.	1. Never<br>2. Rarely<br>3. Sometimes<br>4. Most of the time<br>5. Always
P3_6	Participation in class (answering questions, presentations, etc.)	1. Never<br>2. Rarely<br>3. Sometimes<br>4. Most of the time<br>5. Always

P3_7	Limited time to do the work	1. Never<br>2. Rarely<br>3. Sometimes<br>4. Most of the time<br>5. Always
P3_8	Group mates make faster progress on homework and/or academic work.	1. Never<br>2. Rarely<br>3. Sometimes<br>4. Most of the time<br>5. Always
P4_1	Sleep disturbances (insomnia or nightmares)	1. Never<br>2. Rarely<br>3. Sometimes<br>4. Most of the time<br>5. Always
P4_2	Chronic fatigue (permanent tiredness)	1. Never<br>2. Rarely<br>3. Sometimes<br>4. Most of the time<br>5. Always
P4_3	Headaches or migraines	1. Never<br>2. Rarely<br>3. Sometimes<br>4. Most of the time<br>5. Always
P4_4	Digestion problems, abdominal pain or diarrhoea	1. Never<br>2. Rarely<br>3. Sometimes<br>4. Most of the time<br>5. Always
P4_5	Scratching, nail biting, rubbing, etc.	1. Never<br>2. Rarely<br>3. Sometimes<br>4. Most of the time<br>5. Always
P4_6	Drowsiness or increased need for sleep.	1. Never<br>2. Rarely<br>3. Sometimes<br>4. Most of the time<br>5. Always
P4_7	Muscle aches and/or contractures	1. Never<br>2. Rarely<br>3. Sometimes<br>4. Most of the time<br>5. Always
P4_8	Skin reactions (rash, peeling, etc.)	1. Never<br>2. Rarely<br>3. Sometimes<br>4. Most of the time<br>5. Always
P4_9	Restlessness (inability to relax and be calm)	1. Never<br>2. Rarely<br>3. Sometimes<br>4. Most of the time<br>5. Always

P4_10	Anxiety, distress or despair	1. Never<br>2. Rarely<br>3. Sometimes<br>4. Most of the time<br>5. Always
P4_11	Increased or decreased food intake	1. Never<br>2. Rarely<br>3. Sometimes<br>4. Most of the time<br>5. Always
P4_12	Feelings of depression and sadness (downhearted)	1. Never<br>2. Rarely<br>3. Sometimes<br>4. Most of the time<br>5. Always
P4_13	Feelings of aggression or increased irritability	1. Never<br>2. Rarely<br>3. Sometimes<br>4. Most of the time<br>5. Always
P4_14	Sudden mood swings	1. Never<br>2. Rarely<br>3. Sometimes<br>4. Most of the time<br>5. Always
P4_15	Conflict or tendency to argue or quarrel	1. Never<br>2. Rarely<br>3. Sometimes<br>4. Most of the time<br>5. Always
P4_16	Isolation from others	1. Never<br>2. Rarely<br>3. Sometimes<br>4. Most of the time<br>5. Always
P4_17	Unwillingness to do your work as a student	1. Never<br>2. Rarely<br>3. Sometimes<br>4. Most of the time<br>5. Always
P5_1	Making a plan for the execution of your tasks	1. Never<br>2. Rarely<br>3. Sometimes<br>4. Most of the time<br>5. Always
P5_2	Self-praise	1. Never<br>2. Rarely<br>3. Sometimes<br>4. Most of the time<br>5. Always
P5_3	Venting and confiding (verbalising the situation of concern)	1. Never<br>2. Rarely<br>3. Sometimes<br>4. Most of the time<br>5. Always

P5_4	I tried to get something positive or beneficial out of the stressful situation.	1. Never<br>2. Rarely<br>3. Sometimes<br>4. Most of the time<br>5. Always
P5_5	Taking up a hobby (physical activity, reading, watching series, social networking, etc.)	1. Never<br>2. Rarely<br>3. Sometimes<br>4. Most of the time<br>5. Always
P5_6	Accompanying a loved one (family, pets, friends, etc.).	1. Never<br>2. Rarely<br>3. Sometimes<br>4. Most of the time<br>5. Always
StSIS	Total Stressors SISCO-II	
RfsSIS	Total Physical and Psychological Reactions SISCO-II	
RcsSIS	Total social behavioural reactions SISCO-II	
RxTSIS	Total Total Reaction Total SISCO-II	
Total SIS	Total Academic Stress Measurement SISCO-II	
CopSIS	Total Coping SISCO-II (Factor 4)	
TSIS.Af	Total SISCO-II + coping baseline	
	General data	
TTOPsic	Are you currently in psychological treatment?	1:Yes 0:No
TTOfar	Are you currently on any medication?	1:Yes 0:No
